# Use of Amylomaltase to Steer the Functional and Nutritional Properties of Wheat Starch

**DOI:** 10.3390/foods10020303

**Published:** 2021-02-02

**Authors:** Konstantinos Korompokis, Lomme J. Deleu, Niels De Brier, Jan A. Delcour

**Affiliations:** 1 Laboratory of Food Chemistry and Biochemistry and Leuven Food Science and Nutrition Research Centre (LFoRCe), KU Leuven, Kasteelpark Arenberg 20, B-3001 Leuven, Belgium; lomme.deleu@kuleuven.be (L.J.D.); jan.delcour@kuleuven.be (J.A.D.); 2 Belgian Red Cross, Motstraat 42, B-2800 Mechelen, Belgium; niels.debrier@rodekruis.be

**Keywords:** amylomaltase, wheat starch, amylopectin chain elongation, starch rheological properties, in vitro starch digestion

## Abstract

The fine molecular structure of starch governs its functionality and digestibility, and enzymatic approaches can be utilized to tailor its properties. The aim of this study was to investigate the in situ modification of starch by amylomaltase (AMM) from Thermus thermophilus in model starch systems subjected to hydrothermal treatments under standardized conditions and the relationship between molecular structure, rheological properties and in vitro digestibility. When low dosages of AMM were added to a wheat starch suspension prior to submitting it to a temperature-time profile in a Rapid Visco Analyzer, the increased peak viscosity observed was attributed to partial depolymerization of amylose, which facilitated starch swelling and viscosity development. At higher dosages, the effect was smaller. The low cold paste viscosity as a result of the activity of AMM reflected substantial amylose depolymerization. At the same time, amylopectin chains were substantially elongated. The longer amylopectin chains were positively correlated (*R*^2^ = 0.96) with the melting enthalpies of retrograded starches, which, in turn, were negatively correlated with the extent (*R*^2^ = 0.92) and rate (*R*^2^ = 0.79) of in vitro digestion. It was concluded that AMM has the potential to be used to deliver novel starch functionalities and enhance its nutritional properties.

## 1. Introduction

Starch is the most abundant glycemic carbohydrate in the human diet. In the gastrointestinal tract, it is mainly digested by salivary and pancreatic α-amylase and intestinal mucosal α-glucosidases. Pancreatic α-amylase internally hydrolyzes α-1,4-glycosidic bonds, thereby releasing short-chain hydrolysis products, namely maltose, maltotriose and α-limit dextrins. The latter contain undigested α-1,6-glycosidic bonds [1]. Next, the α-glucosidases on the small intestine epithelium continue the hydrolysis into glucose that is then absorbed by the enterocytes [2]. Undoubtedly, the molecular structure of starch polymers governs starch digestion, especially in the absence of external barriers that impede access/binding of pancreatic α-amylase to starch [3].

Starch consists of two polymers, i.e., amylose (AM) and amylopectin (AP), which differ in susceptibility to digestive α-amylases.

AM is an essentially linear polymer. It consists of glucose units predominantly linked by α-1,4-glycosidic bonds. AP is a highly branched polymer with approximately 5% of its glycosidic bonds being of the α-1,6 type [4]. While one would suspect that the branched nature of the AP and its high molecular weight would facilitate enzymatic hydrolysis [5], the relationship between starch molecular structure and digestibility is far more complex. Starches with high AM content are a good source of enzyme-resistant starch because they tend to retain their granular structure even during hydrothermal processing, and/or quickly re-associate into densely packed double helices thereafter [6]. However, consumption of freshly cooked rice of different varieties but with the same AM content elicited different glycemic responses [7], and in vitro studies have shown that the digestibility of freshly cooked rice starch is impacted by the amount of short AM chains and the total AM molecular size [8]. Multiple molecular aspects thus determine the way AM impacts starch digestion.

Likewise, the molecular structure of AP, and structural aspects such as average chain length and branching density, have a huge impact on starch digestion. The parabolic relationship between (i) the ratio of short to long AP chains of cooked and retrograded maize starch, and (ii) the content of slowly digestible starch, shows that starches dominantly containing either short or long AP chains contain high levels of slowly digestible starch [9]. High content of short branch chains implies higher overall branching density and slower starch digestion due to both the hindrance of pancreatic α-amylase binding in close proximity to a branching point and insufficient saturation of the active subsites [10,11]. Starch with very long AP chains is also slowly digestible, probably because AP chains re-associate upon cooling into dense, crystalline structures that are difficult to be hydrolyzed [9,12]. 

It follows from the above that targeted modification of the structure of starch may reduce the digestibility of starchy foods. Amylomaltase (AMM, E.C. 2.4.1.25) holds potential in this respect. This 4-α-glucanotransferase catalyzes the transfer of a segment of α-1,4-D-glucan to a new 4-position in an acceptor carbohydrate via a disproportionation reaction [13]. When AMM expresses its transferase activity, a cleavage on the donor glucan chain is executed and a covalent enzyme-substrate intermediate complex is formed. Such a complex is then broken down by the acceptor molecule, which accepts the donor glucan [14,15]. AMM from *Thermus thermophilus* is active at temperatures exceeding those at which starch gelatinizes (its temperature of optimal activity is 72 °C [16]) and is an excellent candidate for use during food processing. Starch modification by this AMM leads to complete AM depolymerization and elongation of AP branch chains. It is industrially used for producing thermo-reversible starch gels, as the resultant starches with longer AP chains have excellent gelling capacity [16]. In addition, this AMM displays to a low extent a cyclization activity [16] that leads to cycloamyloses with eight to 32 glucose units [17]. However, taking into account the minimal impact of cyclodextrins on starch pasting properties [18], and the fact that cycloamyloses are easily digestible by pancreatic α-amylase [19], it is expected that their presence only to a limited degree impacts starch functionality.

It is argued here that the enzymatically elongated AP branch chains may be less susceptible to amylolysis. Indeed, modification of pregelatinized maize starch under optimal conditions (70 °C, pH 7.5) for four or more hours by AMM from *Acidothermus cellulolyticus* 11B led to a decrease in rapidly digestible starch content from 80% to 60%, and an increase of slowly digestible starch content from 12% up to 20% [20]. To expand the current knowledge on the potential of AMM use in food production, it is interesting to investigate whether modified starches with novel functionality can be produced in situ during food processing, which evidently avoids time and resource-consuming preincubations. The above prompted us to investigate the in situ modification of starch by AMM in model starch systems supplemented with the enzyme and subjected to hydrothermal treatments under standardized conditions. We thereby focused on the complex relationship between starch molecular structure and its rheological properties and in vitro digestibility. Insights into the effect of AMM on starch functionality and nutritional properties can enhance its utilization in more complex starchy food systems.

## 2. Materials and Methods

### 2.1. Materials and Reagents

Wheat starch was obtained from Tereos Syral (Aalst, Belgium). A liquid preparation of AMM from *Thermus thermophilus* of food grade quality used for producing the food ingredient Etenia™ was kindly provided by Dr Hans Leemhuis (Avebe, Veendam, The Netherlands). All chemicals and reagents were of at least analytical grade and along with porcine pepsin [P6887; 5208 U/mg, units (U) as defined by the manufacturer], porcine pancreatin (P1625; ≥3 × USP specifications) and isoamylase from *Pseudomonas* sp (08124) preparations purchased from Sigma-Aldrich (Bornem, Belgium), unless otherwise specified. 

### 2.2. Amylomaltase Activity

The AMM activity was assayed in triplicate according to van der Maarel et al. [16]. Thus, 0.3 mL 50 mM sodium maleate buffer (pH 6.5) containing 5.0% *w*/*v* maltotriose and 0.1% *w*/*v* maltotetraose was incubated with 0.1 mL properly diluted enzyme preparation in the same buffer for up to 30 min at 70 °C. Samples were then boiled for 30 min to inactivate the enzyme. For each time point (0, 6, 12, 18, 24 and 30 min), a different tube was prepared. The released glucose was measured using a colorimetric assay (Megazyme, Bray, Ireland). One enzyme unit (U) of AMM enzyme activity is defined as the amount of enzyme that under the assay conditions produces 1 μmol of glucose per min at pH 6.5 and 70 °C.

### 2.3. Rheological Properties

The rheological properties of starch suspensions during in situ starch modification by AMM were studied in triplicate with a dose-response approach with a Rapid Visco Analyzer Super 4 (Perten Instruments, Hägersten, Sweden) based on Derde et al. [21]. Wheat starch [10.0% *w*/*w* dry matter (dm)] was suspended in 100 mM sodium maleate buffer (pH 6.0) containing 5.0 mM CaCl_2_ and AMM (0, 0.45, 1.8, 4.5, 9, 18, 27 and 45 U/g starch dm) to obtain 25.0 g final sample weight. The starch suspension was equilibrated at 40 °C for 1 min and the temperature was then increased to 95 °C in 12 min, held constant at 95 °C for 5 min and decreased to 50 °C in 7 min before final isothermal holding at 50 °C for 30 min. The slurries were stirred at 160 rpm throughout analysis. Peak viscosity is the maximum viscosity during heating and the cold paste viscosity that at the end of the rapid visco analysis (RVA) profile. Breakdown viscosity is the difference between peak and minimum viscosity during the 95 °C holding phase. The latter is the hot paste viscosity and the difference between cold and hot paste viscosity is the setback. After the RVA run, the starch slurries were stored at 5 °C for 24 h, frozen with liquid nitrogen and freeze-dried. The dried material was gently ground with mortar and pestle, sieved (aperture: 250 μm) and stored in closed containers at room temperature until further analysis. In addition, for another set of samples used to study the molecular structure of AM and AP, the RVA profile was stopped when the peak viscosity was reached and the starch slurries were immediately frozen with liquid nitrogen and freeze-dried.

### 2.4. High Performance Size Exclusion Chromatography

#### 2.4.1. Starch Debranching

The fine molecular structure of AM and AP was studied as in Dries et al. [22] by using High Performance Size Exclusion Chromatography (HPSEC), i.e., a widely common methodology to study both AM and AP weight chain distributions [23,24]. Sample (10 mg) in deionized water (5.0 mL) in an air-tight glass tube was incubated at 100 °C in a water bath for 1 h. After cooling to room temperature, 1.0 mL of the suspension was transferred to another glass tube and 50 μL 600 mM sodium acetate buffer (pH 4.0) was added prior to adding 12.5 μL isoamylase preparation containing 900 U (U as defined by the supplier). The mixture was incubated for 24 h at 40 °C. The same amount of isoamylase solution was then added once more and the incubation continued for an additional 24 h [22]. Finally, the mixture was boiled for 15 min to inactivate the enzyme, filtered (Millex-HP, 0.45 μm, PES; Millipore, Carrigtwohill, Ireland) and immediately analyzed by HPSEC.

#### 2.4.2. Chromatographic Separation

The chain length distributions of debranched AM and AP samples were studied with HPSEC as described by Dries et al. [22]. Debranched samples (50 μL) were injected (SIL-HTC Auto sampler, Shimadzu, Kyoto, Japan) onto three sequential TSK gel columns (G6000 PWXL, G4000 PWXL and G3000 PWXL) (Tosoh, Stuttgart, Germany) attached to a modular Shimadzu SIL-HTc unit equipped with a pump (LC-20AT), an online degasser (DGU-20A5), a differential refractive index (RI) detector (10A RID, Shimadzu) at 40 °C and a column oven (CTO-20A, Shimadzu) at 60 °C. The flow rate was 0.5 mL/min and the elution medium was 29 mM sodium acetate buffer (pH 4.0). Solutions of Shodex P-82 pullulan standards (1.0 mg/mL, Showa Denko, Tokyo, Japan) were injected (50 μL) to produce a calibration curve. Their molecular weights (g/mol) and corresponding degrees of polymerization (DP) were: 342 (DP 2), 1.32 × 10^3^ (DP 8), 6.2 × 10^3^ (DP 38), 10.0 × 10^3^ (DP 61), 23.0 ×10^3^ (DP 141), 48.8 × 10^3^ (DP 301), 113.0 × 10^3^ (DP 697), 200 × 10^3^ (DP 1234), 348 × 10^3^ (DP 2148) and 805 × 10^3^ (DP 4969). The logarithm of the molecular weights of the standards versus their corresponding elution volumes were plotted and fitted with a third order polynomial equation (R^2^ = 0.999). The first (DP < 100) and second (DP 100–10,000) peak of the weight distributions corresponded to debranched AP and debranched AM, respectively [24]. Furthermore, the weight distributions of the debranched starches were normalized to the maximum of the first peak of the AP distribution (AP1) which represents the short AP branches (DP < 30) [25]. 

### 2.5. Extent of Starch Retrogradation

The extent of retrogradation of starch slurries first prepared in the RVA and then stored for 24 h at 5 °C was studied by differential scanning calorimetry (DSC) with a Q2000 DSC (TA Instruments, New Castle, DE, USA) instrument. The samples were first frozen with liquid nitrogen and freeze-dried. Freeze-dried starch samples were accurately weighed (2.0–3.0 mg) in aluminum pans (Hitachi High-Technologies, Tokyo, Japan) and water [1:5 (*w*/*w*) sample dm:water] was added. Pans were hermetically sealed and left at room temperature for 45 min. They were then further equilibrated at 0 °C before being heated from 0 to 120 °C at 4 °C/min. The peak temperatures [T_p_ (°C)] and enthalpies [ΔH (J/g starch dm)] of the melting of AP crystals formed as a result of retrogradation were determined with the TA Universal Analysis software.

### 2.6. In Vitro Starch Digestion

The in vitro starch digestion assay was based on Korompokis et al. [26]. An aliquot (100 mg) of freeze-dried starch obtained after each RVA run and then stored at 5 °C for 24 h was accurately weighted in flat-bottomed glass tubes, suspended in 6.0 mL deionized water and equilibrated at 37 °C for 15 min. Next, 5.0 mL 20 mM HCl containing 1.0 mg/mL pepsin preparation was added to the suspensions. After 30 min of incubation at 37 °C under magnetic stirring (200 rpm), 5.0 mL 20 mM NaOH was added to cease pepsin activity. Next, 5.0 mL 200 mM sodium acetate buffer (pH 6.0) containing pancreatin preparation (0.5 mg/mL), CaCl_2_ (200 mM) and MgCl_2_ (0.49 mM) was added. The digestion mixture was incubated for 90 min at 37 °C under magnetic stirring (200 rpm). At predetermined time points, 0.1 mL aliquots were transferred into Eppendorf tubes containing 0.2 mL 300 mM sodium carbonate to terminate starch digestion. Following shaking and centrifugation (10,000 × *g*, 10 min, 21 °C), the reducing sugar contents in the supernatants were quantified by the method of Moretti and Thorson [27] using the reagent p-hydrobenzoic hydrazide and a maltose calibration curve (0.0–4.0 mg/mL). The digested starch was thus expressed in maltose equivalent released (%).

The different digestion curves were fitted to a first-order equation as described by Goñi et al. [28] in Equation (1):

Ct = C∞ (1 − e^−kt^) (1)

with C_t_: the percentage of starch digested (%), t: incubation time (min), C_∞_: percentage of starch digested (%) at infinite time and k: rate constant (min^−1^). For fitting purposes, the baseline value at *t* = 0 min was subtracted from the subsequent values at t time. The fitting was performed with the statistical software JMP Pro 14.0 (SAS, Institute, Cary, NC, USA). 

### 2.7. Statistical Analysis

Statistical significance of differences between mean values was assessed by one-way analysis of variance (ANOVA) followed by post hoc Tukey’s test. The confidence level was 95% and the statistical analysis was performed with JMP Pro 14.0 (SAS, Institute, Cary, NC, USA). 

## 3. Results

### 3.1. Impact of Amylomaltase on Starch Rheological Behavior

**Figure 1:**
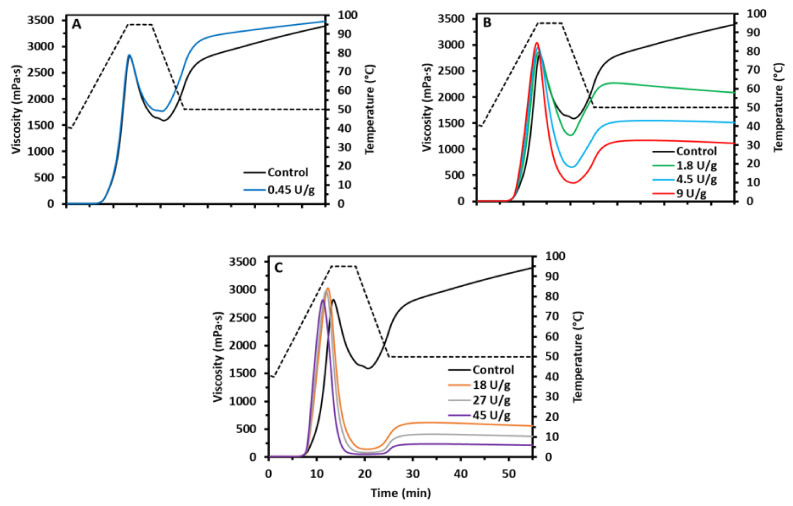
Rapid visco analysis (RVA) profiles of starch suspensions [10.0% *w/w* dry matter (dm)] supplemented with different dosages of amylomaltase (AMM): (**A**) 0 (control) and 0.45, (**B**) 0, 1.8, 4.5 and 9 and (**C**) 0, 18, 27 and 45 U/g starch dm. The temperature profile is denoted with a dotted line.

Different dosages of AMM influenced the viscosity of starch slurries differently. Three AMM dosage categories were considered: low (0.45 U/g starch dm), intermediate (1.8–9 U/g starch dm) and high (18–45 U/g starch dm) (Figure 1 and Table 1). 

The low dosage (Figure 1A) did not impact peak viscosity, yet significantly higher hot paste viscosity and lower breakdown and setback viscosities were recorded. The cold paste viscosity did not significantly increase. Intermediate dosages (Figure 1B) significantly increased peak viscosity. The highest peak viscosity (3050 mPa·s) was recorded at 9 U/g starch dm and was 7.9% higher than that of the control. At the same time, the peak viscosity was reached sooner when the enzyme concentration was increased. The hot paste, breakdown, setback and cold paste viscosities were significantly decreased in a dose-dependent manner. At high dosages (Figure 1C), there was a minor increase in peak viscosity compared to the control and a continuous decrease of hot paste, cold paste and setback viscosities to very low values. It was remarkable that even though the control sample (2825 mPa·s) and that treated with 45 U/g starch dm exhibited the same peak viscosity (2815 mPa·s), their RVA profiles and the rate of viscosity development were explicitly different (Figure 1C).

### 3.2. Amylose and Amylopectin Molecular Structure

**Figure 2:**
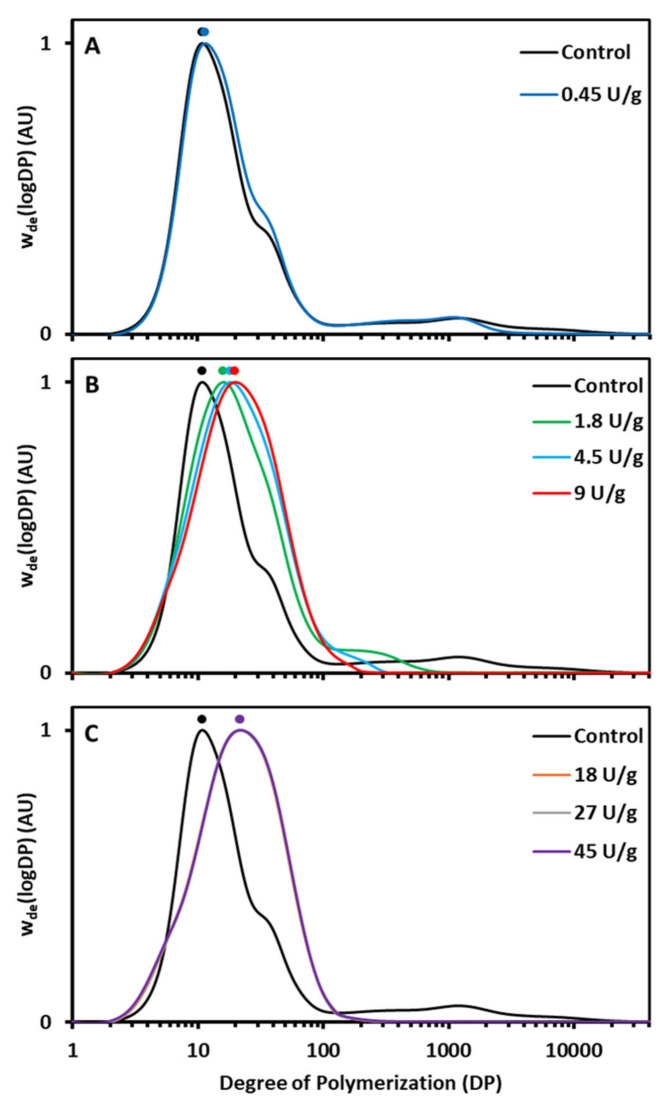
High performance size exclusion chromatography (HPSEC) weight distributions of debranched starches modified by different dosages of amylomaltase (AMM) during rapid visco analysis (RVA): (**A**) 0 (control) and 0.45, (**B**) 0, 1.8, 4.5 and 9 and (**C**) 0, 18, 27 and 45 U/g starch dm. Normalized weight distributions are expressed in arbitrary units (AU). The dots represent the degree of polymerization (DP) values at peak maximum.

Analysis of the branches of control and AMM treated starches for unmodified native wheat starch revealed two characteristic distributions in the HPSEC profiles (Figure 2). The first (DP 2–100) and second (DP 100–10,000) peaks corresponded to debranched AP and debranched AM, respectively [24]. The debranched AP peak showed a maximum at DP 11 (AP1) and a shoulder (AP2) at about DP 35. The AM peak maximum was at around DP 1200. Such distribution is typical for wheat starch when studied with HPSEC [23,24].

While the low AMM concentration only had a minor effect on the AP chain length distribution, it did result in considerable shortening of the long AM chains (DP range decreased to a range of 100–2500) (Figure 2A). At intermediate concentrations, the impact on both polymers was more evident (Figure 2B). There was a gradual shift of the AP chain distributions towards higher DP values, while the characteristic shoulder at DP 35 could no longer be distinguished. Clearly, the portion of AP chains with DP 20–100 substantially increased. In parallel, the AM peak shifted to lower DP values, and with increasing AMM dosages, it gradually disappeared. At the high enzyme concentration, there was no additional effect on the distribution of the AP chains while the AM peak completely disappeared (Figure 2C). 

### 3.3. Extent of Starch Retrogradation

Control starch and starch modified with the low AMM dosage resulted in low ΔH (about 0.4 to 0.8 J/g starch dm) readings (Table 1). At intermediate dosages, the ΔH values gradually increased to 5.6 J/g starch dm. At the high dosages, there was a modest additional effect over that exerted by intermediate dosages. In this case, the ΔH readings reached values as high as 7.7 J/g starch dm. It is noteworthy that the T_p_ readings drastically increased with AMM dosages. While T_p_ was 50 °C for the control starch, T_p_ readings were higher (i.e., up to 70 °C) for AMM treated samples.

### 3.4. In Vitro Starch Digestibility

**Figure 3:**
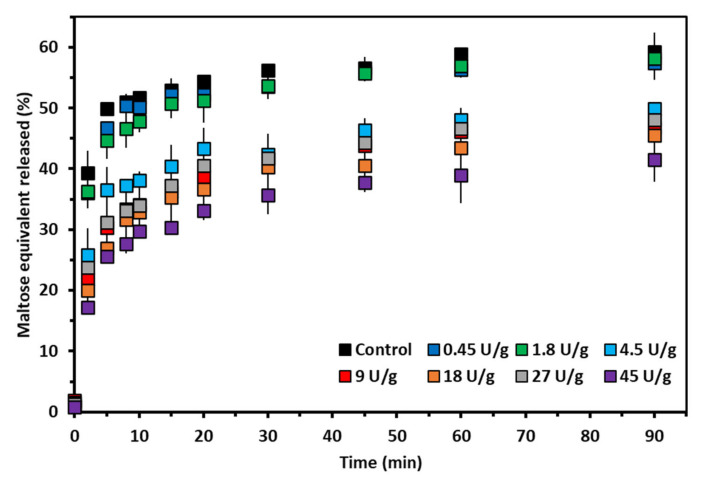
Digestion curves of starches modified by different dosages of amylomaltase (AMM) (U/g starch dm) during rapid visco analysis (RVA) and subsequently stored at 5 °C for 24 h.

Figure 3 shows the digestion curves of the starches first modified by AMM during an RVA run and then stored for 24 h at 5 °C. Prior treatment with AMM considerably reduced starch digestibility. Control starch and starch treated with the low AMM dosage were digested at similar rates and to similar extents (Table 2). Both the extent and the rate of starch digestion substantially declined at intermediate AMM dosages (up to 35% and 62%, respectively), while there was almost no additional effect on starches treated with the high dosages. 

## 4. Discussion

### 4.1. Amylomaltase Induced Modifications of Starch Structure 

AMM action during the RVA run had a profound impact on AM and AP molecular structure. The AM population was gradually eliminated and the AP population increasingly had longer chains (Figure 2). AMM can very effectively transfer AM segments to AP chains leading to their elongation and simultaneous disappearance of the former [16]. In the present case, a low enzyme dosage shortened AM chains to a limited extent, while intermediate dosages led to elongated AP chains and very short residual AM chains. When high dosages were applied, AM was completely degraded and hence no further AP chain elongation occurred. In order to validate that the longer chains in the region of debranched AP in the HPSEC elution profile actually originated from AP and were not hydrolysis products of AM released by AMM, some samples were also run without the debranching treatment (Figure S1). It was found that no short-chain hydrolysis products originated from AM as a result of AMM activity.

Starch structure was not only studied for samples withdrawn at the end of the RVA run but also when the viscosity reached its peak. Already at this point in the RVA run were the HPSEC profiles similar (Figure S2) to those of the samples withdrawn at the end (Figure 2). This illustrates that under the applied conditions AMM acted rather fast and that only small, if any, additional structural changes occurred during the 95 °C holding phase and onwards. Thus, AMM in situ modified the molecular structure of starch polymers during hydrothermal processing of native starch to degrees that depended on the applied dosages. 

### 4.2. Links between In Situ Starch Modification by Amylomaltase and the Resultant Changes in Rheological Behavior 

In this study, a model starch system was used to study the potential of AMM to modify starch in situ during hydrothermal processing. To some extent, doing so mimicked food processing conditions wherein enzymes are included in the initial ingredient mix and act when starch gelatinizes during heating. At the same time, while such conditions do not fully represent the conditions for producing a whole range of various starch-containing food products, similar model systems have been effectively utilized in studies of the enzyme functionality in bread systems [21,29,30].

The viscosity of the starch slurries was clearly affected by in situ action of AMM in a dose-dependent way. The low enzyme dosage led to higher hot paste, lower breakdown and setback and moderately higher cold paste viscosity (Figure 1A). Partially shortened AM chains (Figure 2A) better stabilized the starch slurries and led to stronger gels. The present observations are in accordance with previous work on enzymatically trimmed AM chains in which higher end viscosities were reached due to higher mobility and enhanced AM—AM interactions [29]. Stronger AM association may also contribute to higher hot paste viscosity, as AM minimizes shear-induced breakdown [31]. 

Further modifications at intermediate enzyme dosages led to improved swelling and viscosity development but also to weaker gels (Figure 1B). To the best of our knowledge, ours is the first report to find that starch peak viscosity can be increased as a result of in situ supplementation of a starch modifying enzyme, let alone that it would have been shown for AMM. This increase is mainly attributed to a gradual decrease of the AM content and chain length, and to lesser extent to AP chain elongation. Indeed, AM is generally considered to restrict starch swelling [32]. It is, therefore, logical to assume that selective chain shortening of AM by AMM (Figure 2B) enhances starch swelling and, subsequently, leads to higher peak viscosities. It has been shown earlier that rice AM content is negatively correlated with peak viscosity [31] and that shorter AM chains lead to higher peak viscosity [33]. However, elongated AP chains may also lead to higher peak viscosity. Indeed, a higher portion of longer (DP ≥ 35) AP chains has been correlated with enhanced swelling in normal wheat starches [34]. Next to the increase in peak viscosity, there was also a drastic decrease of the hot and cold paste viscosities. The short AM chains could not prevent shear-induced breakdown and evidently did not allow developing a continuous starch gel network during the RVA cooling phase. 

When high enzyme dosages were used, there was a minor increase in the peak viscosity and a continuous decrease in hot paste, cold paste and setback viscosities. Peak viscosities were reached earlier as a result of the enzyme action (Figure 1C). The higher rate of starch swelling and viscosity development can be attributed to instant and complete breakdown of accessible AM (mainly after gelatinization), which would otherwise have restricted starch swelling. Evidently, completely degraded AM (Figure 2C) no longer contributed to gel formation and the end viscosity could be attributed to residual swollen starch granules solely composed of AP. Here, an analogy with waxy starch can be considered. Waxy starches exhibit higher and earlier peak viscosity than their normal counterparts and lead to lower end viscosities. This has been attributed to the absence of AM that otherwise would restrict swelling and be the building block of the starch gel structures during cooling [35]. In the present study, the elongated AP chains were not long and/or abundant enough to entangle, form gel networks and compensate for the loss of AM. 

The decreased peak viscosities after the use of high AMM dosages compared to those resulting from the use of intermediate AMM dosages may indicate more extensive AP degradation as a whole. Indeed, amylolytic activity has been reported at high AMM dosages [16]. Once AM is depleted, AP serves both as substrate and acceptor, which leads to AP which is smaller in size even if it contains elongated chains [17]. Amylolytic endo-action on AP molecules lowers peak viscosity as also shown by Leman et al. [29]. In conclusion, when the enzyme dosages were high, AM was broken down at such high rates that swelling during gelatinization occurred faster and to a higher extent, while leaving no AM to form a gel upon cooling.

Overall, the impact of different AMM dosages on the viscosity of starch slurries could be directly related to the extent of AM structure modification. Low dosages shortened AM chains to some extent and thus made them more mobile. This positively impacted hot and cold paste viscosities due to enhanced AM—AM interactions. At intermediate levels, the effect on AM depletion was more dominant. This led to more drastic changes in rheological behavior and highlighted the role of AM in starch swelling and viscosity development. High levels of AMM did not induce further changes in the molecular structure of starch polymers and consequently, only had limited additional impact. However, they did result in an increased rate of swelling and viscosity development. 

### 4.3. Retrogradation of Amylomaltase Modified Starch

When the modified starches were stored at 5 °C for 24 h, AP retrograded and thus formed crystalline structures. However, the extent of retrogradation varied with the extent of enzyme induced AP modification (Table 1). In particular, at low dosage, no changes in the ΔH and T_p_ readings were detected due, reasonably, to (hardly) unaltered AP structures. The use of intermediate dosages resulted in both drastically increased ΔH and T_p_ values. Starch with elongated AP chains has a higher propensity and/or potential to retrograde. As also reported by Hansen et al. [36], the formed crystals are also more resistant to melting, as temperatures of up to 70 °C were required to do so. At high dosages, the additional impact on retrogradation over that noted at intermediate dosages was rather limited, since, as noted above, no additional AP modification occurred. The melting enthalpies of retrograded AP in rice gels containing longer AP chains as a result of enzymatic treatment were also significantly higher than those noted for control gels [37]. In the present case, the higher relative amount of longer chains was reflected in a shift of the AP peak in HPSEC profiles towards higher DP values and an increase of DP values of the peak maximum (AP1) (Figure 2). The increased DP values of the peak maximum (AP1) were positively and strongly (*R*^2^ = 0.96) correlated to the ΔH values (Figure S3). It was apparent then that elongated AP chains form crystalline structures which are more stable and thus melt at higher temperatures.

### 4.4. In Vitro Enzymatic Susceptibility of Starch In Situ Modified by Amylomaltase

Starch susceptibility to amylolysis in an in vitro digestion assay was largely impacted by the extent of starch modification by AMM. At the low enzyme concentration, there were no detectable changes in the extent and rate of starch digestion because no major modification of AP fine structure occurred (Figure 2A). In this case, shortening of AM chains length did not have an impact on overall starch digestibility. 

**Figure 4:**
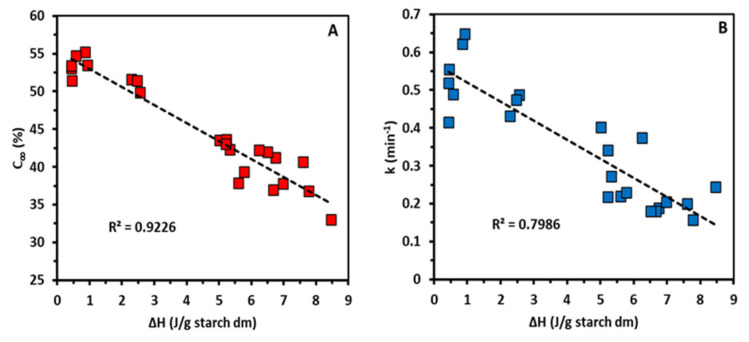
Relationship between the melting enthalpies (ΔH) of starches modified by different dosages of amylomaltase (AMM) (U/g starch dm) during rapid visco analysis (RVA) and subsequently stored at 5 °C for 24 h and (**A**) the extent of starch digestion (C_∞_) and (**B**) the starch digestion rate constant (k).

At intermediate concentrations, both the extent and rate of digestion significantly declined (Table 2). That could clearly be linked to elongation of AP chains as confirmed by a negative correlation between the DP values of the peak maximum (AP1) and C_∞_ (R^2^ = 0.91) and k (*R*^2^ = 0.93), respectively (Figure S3). Higher portions of long AP chains lead to slower starch digestion probably only when retrogradation occurs upon cold storage (e.g., at 4 °C overnight) [9,12], since no impact of AP chain length was shown for freshly gelatinized starch [25]. Earlier, starch containing AP chains elongated by amylosucrase was also demonstrated to form crystalline structures with elevated ΔH values and lower starch digestibility than those of the unmodified counterpart [38,39]. In the present study, the negative correlations between ΔH and C_∞_ (Figure 4A) and ΔH and k (Figure 4B) showed that AMM treated starch is digested to a lower extent and at a lower rate than control starch due to the formation of enzyme resistant crystalline arrangements. Therefore, elongation of AP chains by AMM induced higher extent of retrogradation, which was reflected in a lower rate and extent of starch digestion. Starch retrogradation cannot only lower the extent but also the catalytic efficiency of pancreatic α-amylase, and thus also the rate of starch digestion [40]. Comparison of the post-prandial glycemic responses resulting from the consumption of freshly boiled potatoes or such potatoes subsequently cold stored (8 °C for 24 h) has revealed that retrograded starch also elicits lower post-prandial glycemic responses [41]. 

Upon digestion, pancreatic α-amylase randomly cleaves α-1,4-glycosidic bonds in an endoactivity pattern and releases starch chains of various DP which are further completely digested to release maltose, maltotriose and α-limit dextrins [1]. In this context, it is of note that during in vitro digestion of high-AM maize starches, the released linear AM chain segments in the first stages of the digestion reassociate into crystalline structures that resist digestion. Such enzyme resistant structures contain 13 to 30 glucose units [42,43]. Witt et al. [43] speculated that these linear segments not only originate from the digestion of AM, but also from that of AP. In the present study, the modified starches had a relatively higher content of AP chains with DP 20–100 (Figure 2). It is plausible, then, that the digestion of these elongated AP chains respectively released longer hydrolysis products compared to the control. Therefore, fragments of the elongated AP chains generated in situ during digestion may have even higher propensity to aggregate during digestion and become resistant to amylolysis than released AP chains of unmodified starch.

Lastly, at high dosage there was no additional effect on starch digestibility compared to that resulting from the intermediate dosage. This was expected since there were no major additional modifications on AP structure governing its ability to form crystalline structures and, thus, to lower starch digestibility. The slight amylolytic activity had no effect on starch digestibility. 

It is clear from the above that the extent of AP chain length modification by AMM governs starch digestibility. At low AMM dosage, there was no significant impact on AP structure, and consequently, only a limited impact on starch digestion. At intermediate dosages, elongated AP chains were more abundant and formed more and/or more easily crystalline structures upon cold storage at 5 °C for 24 h, and starch digestibility was evidently decreased. Since the high dosages had no additional effect on AP structure, no additional impact on starch digestion was observed. From a nutritional point of view, then, adequately chosen AMM dosages can sufficiently elongate AP chains and lower starch digestibility.

## 5. Conclusions

**Figure 5:**
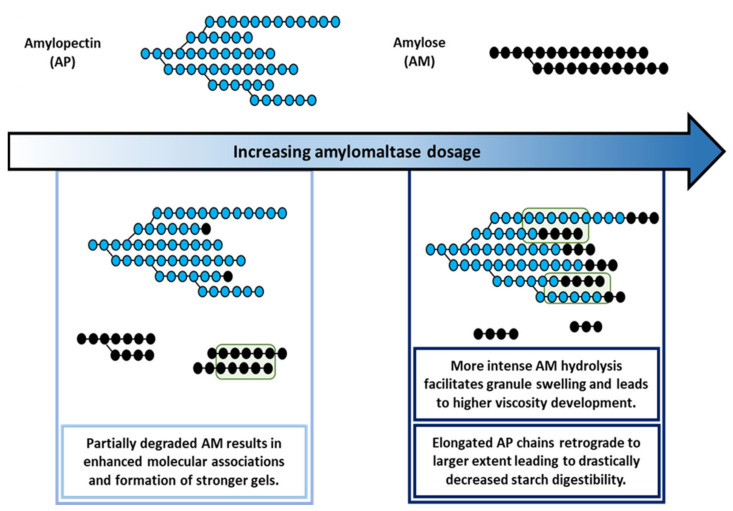
Schematic illustration of the molecular structures of amylose (AM) and amylopectin (AP) obtained by amylomaltase (AMM) treatment with various dosages during rapid visco analysis (RVA). Blue and black circles represent AP and AM building units, respectively. The schematic representation does not reflect the relative molecular weights and sizes of the two polymers. The green box illustrates the enhanced interactions between polymers.

In this work, the impact of various AMM dosages on the molecular structure of starch and the subsequent effect on starch rheological properties and digestibility was studied for the first time. As schematically presented in (Figure 5), as a result of AMM activity, AM was gradually depolymerized and used as a substrate for the elongation of AP chains. For low dosages, AM was partially depolymerized, which enhanced its potential to form strong gels during cooling. At intermediate dosages, AM was depolymerized to a larger extent. This gave rise to higher peak viscosity due to enhanced starch swelling. However, this effect was attenuated at high AMM dosages. The gradual depolymerization up to the complete disappearance of AM reduced the capacity to form gels during cooling. At the same time, the elongated AP chains had a considerably higher propensity to form crystalline structures during storage at 5 °C for 24 h. Such structures were digested more slowly and to a lower extent compared to what was noted for unmodified starch.

In conclusion, the use of AMM induces novel starch functionality and the considerable decrease in starch digestibility imparts AMM with a great application potential. The holistic approach in this study gives a basic understanding of how different AMM dosages can variably influence the physicochemical properties and digestibility of starch. Insights from this fundamental study can be of great use in the context of the application of AMM for producing starch-containing foods. AMM can thus be utilized in the production of a range of starchy food products as a processing agent to manipulate viscosity when deemed useful. Furthermore, AMM can be used in the production of starchy foods with lower starch digestibility and sustained glucose release that provide the consumer with healthier options. The use of starch-modifying enzymes in foods with the goal of lowering their glycemic potential is not commonly practiced in food industry. Hence, knowledge on enzymes such as AMM can contribute to novel approaches to improve food nutritional properties without compromising quality.

## Figures and Tables

**Table 1:** Rapid visco analysis (RVA) properties of starch suspensions [10.0% *w/w* starch dry matter (dm)] treated with different dosages of amylomaltase (AMM, U/g starch dm) and melting enthalpies (ΔH) and peak melting temperatures (T_p_) of the modified starches after storage at 5 °C for 24 h.

Enzyme Concentration(U/g starch dm)	Viscosity (mPa·s)						
	Peak	Hot Paste	Cold Paste	Breakdown	Setback	ΔH (J/g starch dm)	T_p_ (°C)
0	2825 (40) ^a^	1590 (10) ^a^	3395 (30) ^a^	1230 (20) ^e^	1800 (20) ^a^	0.78 (0.18) ^e^	49.60 (0.76) ^d^
0.45	2845 (25) ^a^	1770 (15) ^b^	3485 (50) ^a^	1075 (10) ^f^	1715 (35) ^b^	0.44 (0.01) ^e^	50.21 (1.11) ^d^
1.8	2860 (40) ^a^	1270 (40) ^c^	2090 (60) ^b^	1560 (70) ^d^	820 (25) ^c d^	2.39 (0.14) ^d^	63.82 (3.36) ^c^
4.5	2955 (40) ^b^	660 (80) ^d^	1515 (60) ^c^	2300 (60) ^c^	855 (20) ^c^	5.18 (0.16) ^c^	66.05 (0.65) ^bc^
9	3050 (45) ^b^	355 (35) ^e^	1115 (60) ^d^	2695 (60) ^b^	760 (30) ^d^	5.57 (0.34) ^b c^	67.11 (0.88) ^abc^
18	3040 (40) ^b^	140 (15) ^f^	560 (45) ^e^	2900 (10) ^a^	420 (30) ^e^	6.40 (0.53) ^bc^	68.08 (0.39) ^ab^
27	2985 (20) ^b^	80 (5) ^f^	370 (25) ^f^	2905 (10) ^a^	290 (20) ^f^	6.78 (0.72) ^ab^	68.96 (0.23) ^ab^
45	2815 (20) ^a^	50 (5) ^f^	210 (5) ^g^	2765 (20) ^b^	160 (10) ^g^	7.73 (0.74) ^a^	70.35 (0.46) ^a^

Standard deviations are given between brackets. Mean values (*n* = 3) within a column with different superscript letters were significantly different (*p* < 0.05; Tukey’s test).

**Table 2:** Kinetic parameters (C_∞_ and k) obtained by fitting the experimental data on first-order kinetics for the starches modified by different dosages of amylomaltase (AMM) (U/g starch dm) during rapid visco analysis (RVA) and subsequently stored at 5 °C for 24 h.

Enzyme Concentration (U/g starch dm)	C_∞_ (%)	k (min^−1^)
0	54.5 (0.9) ^a^	0.59 (0.09) ^a^
0.45	52.6 (1.1) ^a^	0.50 (0.07) ^ab^
1.8	50.9 (0.9) ^a^	0.46 (0.03) ^ab^
4.5	43.2 (0.7) ^b^	0.33 (0.06) ^bc^
9	40.4 (3.6) ^bc^	0.22 (0.01) ^c^
18	39.2 (2.1) ^bc^	0.20 (0.02) ^c^
27	41.6 (0.8) ^b^	0.24 (0.10) ^c^
45	35.9 (2.5) ^c^	0.20 (0.04) ^c^

C_∞_: percentage of starch digested (% of dm) at infinite time; k: rate constant. Standard deviations are given between brackets. Mean values (*n* = 3) within a column with different superscript letters were significantly different (*p* < 0.05; Tukey’s test).

## Data Availability

Data available on request.

## Supplementary Materials

The following are available online at https://www.mdpi.com/2304-8158/10/2/303/s1, Figure S1: Two examples of high performance size exclusion chromatography (HPSEC) weight distributions of intact (not subjected to de-branching treatment; black line) and debranched starches (red line) modified by different dosages of amylomaltase (AMM) during rapid visco analysis (RVA): (A) 0 (control) and (B) 45 U/g starch dm. Weight distributions are expressed in arbitrary units (AU) and were not normalized, Figure S2: High performance size exclusion chromatography (HPSEC) weight distributions of debranched starches modified by different dosages of amylomaltase (AMM) during rapid visco analysis (RVA): (A) 0 (control), (B) 0.45, (C) 1.8, (D) 4.5, (E) 9, (F) 18, (G) 27 and (H) 45 U/g starch dm. Normalized weight distributions are expressed in arbitrary units (AU). Samples were collected either at the point of peak viscosity development or at the end of the RVA profile; Figure S3: Linear relationship between the degree of polymerization (DP) of the peak maximum of debranched amylopectin and (A) extent of digestion (C_∞_), (B) digestion rate (k) and (C) melting enthalpies (ΔH) of starches first modified by different dosages of amylomaltase (AMM) during rapid visco analysis (RVA) and subsequently stored at 5 °C for 24 h.
