# Supplementary material for: Use of Amylomaltase to Steer the Functional and Nutritional Properties of Wheat Starch
Source: Foods. 2021 Feb 2;10(2):303. doi: 10.3390/foods10020303 (PMC7913068; doi:10.3390/foods10020303)
Supplement: Supplementary file 1 [file foods-10-00303-s001.pdf]

foods

MDPI

*Article*

# Supplementary material

# Use of amylomaltase to steer the functional and nutritional properties of wheat starch

**Konstantinos Korompokis[1]*, Lomme J. Deleu[1], Niels De Brier[2] and Jan A. Delcour[1]**

[1] Laboratory of Food Chemistry and Biochemistry and Leuven Food Science and Nutrition Research Centre (LFoRCe), KU Leuven, Kasteelpark Arenberg 20, B-3001 Leuven, Belgium; lomme.deleu@kuleuven.be (L.J.D.); jan.delcour@kuleuven.be (J.A.D.)
[2] Belgian Red Cross, Motstraat 42, B-2800 Mechelen, Belgium; niels.debrier@rodekruis.be
* Correspondence: konstantinos.korompokis@kuleuven.be; Tel.: +32-163-22-783

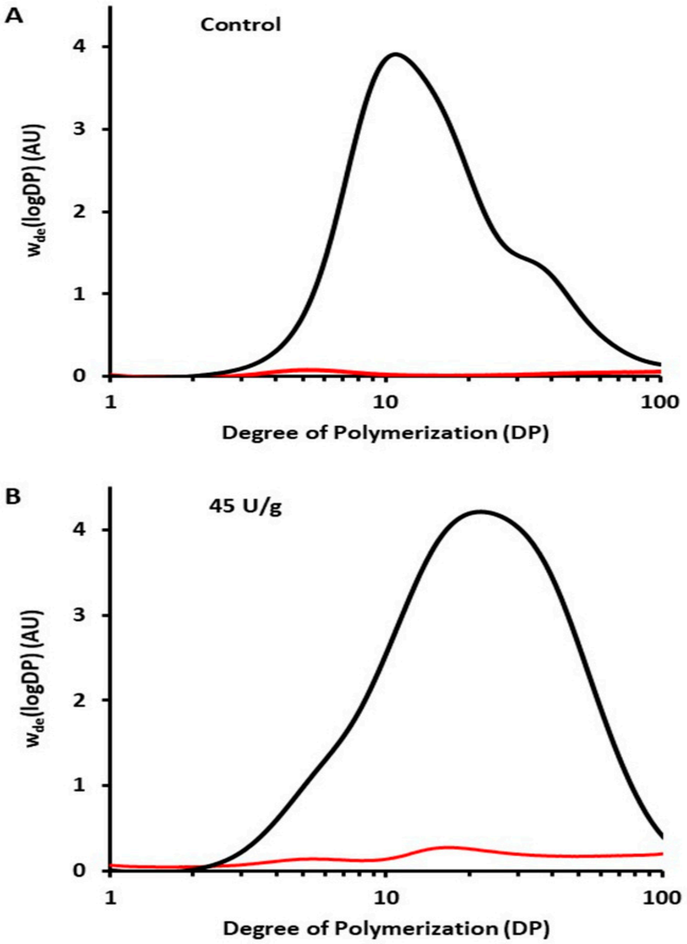


**Figure S1.** High performance size exclusion chromatography (HPSEC) weight distributions of intact (not subjected to debranching treatment; black line) and debranched starches (red line) modified by different dosages of amylomaltase (AMM) during rapid visco analysis (RVA): (**A**) 0 (control) and (**B**) 45 U/g starch dm. Weight distributions are expressed in arbitrary units (AU) and were not normalized.

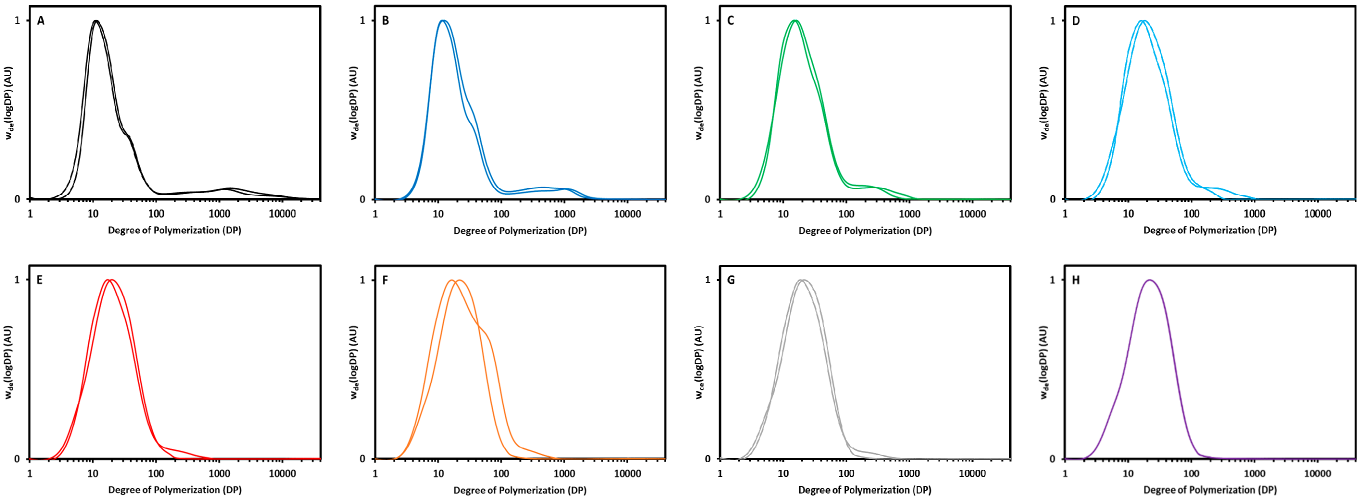


**Figure 2.** High performance size exclusion chromatography (HPSEC) weight distributions of debranched starches modified by different dosages of amylomaltase (AMM) during rapid visco analysis (RVA): (**A**) 0 (control), (**B**) 0.45, (**C**) 1.8, (**D**) 4.5, (**E**) 9, (**F**) 18, (**G**) 27 and (**H**) 45 U/g starch dm. Normalized weight distributions are expressed in arbitrary units (AU). Samples were collected either at the point of peak viscosity development or at the end of the RVA profile.

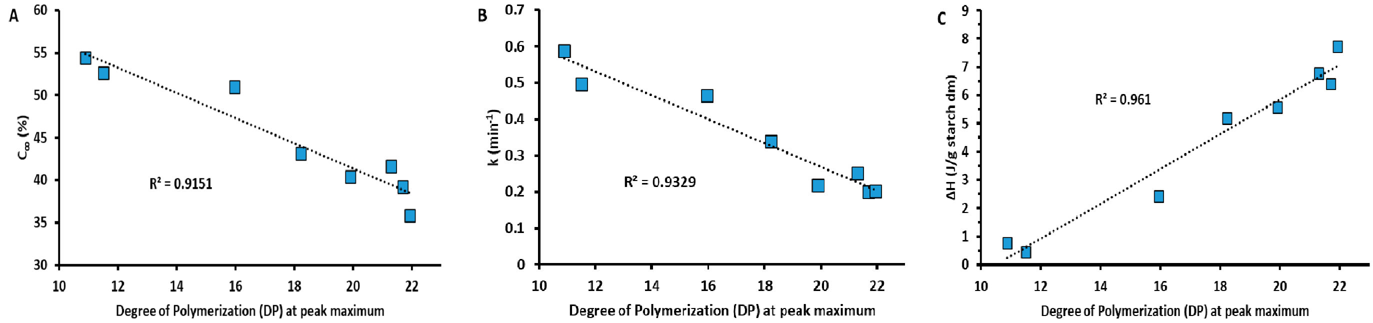


**Figure 3.** Linear relationship between the degree of polymerization (DP) of the peak maximum of debranched amylopectin and (**A**) extent of digestion (C∞), (**B**) digestion rate (k) and (**C**) melting enthalpies (ΔH) of starches first modified by different dosages of amylomaltase (AMM) during rapid visco analysis (RVA) and subsequently stored at 5 °C for 24 h.
